# A short peptide exerts neuroprotective effects on cerebral ischemia–reperfusion injury by reducing inflammation via the miR-6328/IKKβ/NF-κB axis

**DOI:** 10.1186/s12974-023-02739-4

**Published:** 2023-02-28

**Authors:** Yilin Li, Tao Jin, Naixin Liu, Junsong Wang, Zihan Qin, Saige Yin, Yingxuan Zhang, Zhe Fu, Yutong Wu, Yinglei Wang, Yixiang Liu, Meifeng Yang, Ailan Pang, Jun Sun, Ying Wang, Xinwang Yang

**Affiliations:** 1 Department of Anatomy and Histology and Embryology, Faculty of Basic Medical Science, Kunming Medical University, Kunming, 650500 Yunnan China; 2 Key Laboratory of Chemistry in Ethnic Medicinal Resources and Key Laboratory of Natural Products Synthetic Biology of Ethnic Medicinal Endophytes, State Ethnic Affairs Commission and Ministry of Education, School of Ethnic Medicine, Yunnan Minzu University, Kunming, 650504 Yunnan China; 3 Department of Orthopedics, 920th Hospital of Joint Logistics Support Force of PLA, Kunming, 650032 Yunnan China; 4 Department of Neurology, First Affiliated Hospital of Kunming Medical University, Kunming, 650031 Yunnan China

**Keywords:** Ischemic stroke, Peptide, miR-6328, Neuroprotection, IKKβ, NF-κB

## Abstract

**Background:**

Despite considerable efforts, ischemic stroke (IS) remains a challenging clinical problem. Therefore, the discovery of effective therapeutic and targeted drugs based on the underlying molecular mechanism is crucial for effective IS treatment.

**Methods:**

A cDNA-encoding peptide was cloned from RNA extracted from *Rana limnocharis* skin, and the mature amino acid sequence was predicted and synthesized. Hemolysis and acute toxicity of the peptide were tested. Furthermore, its neuroprotective properties were evaluated using a middle cerebral artery occlusion/reperfusion (MCAO/R) model in rats and an oxygen–glucose deprivation/reperfusion (OGD/R) model in neuron-like PC12 cells. The underlying molecular mechanisms were explored using microRNA (miRNA) sequencing, quantitative real-time polymerase chain reaction, dual-luciferase reporter gene assay, and western blotting.

**Results:**

A new peptide (NP1) with an amino acid sequence of ‘FLPAAICLVIKTC’ was identified. NP1 showed no obvious toxicities in vivo and in vitro and was able to cross the blood–brain barrier. Intraperitoneal administration of NP1 (10 nmol/kg) effectively reduced the volume of cerebral infarction and relieved neurological dysfunction in MCAO/R model rats. Moreover, NP1 significantly alleviated the decrease in viability and increase in apoptosis of neuron-like PC12 cells induced by OGD/R. NP1 effectively suppressed inflammation by reducing interleukin-1β (IL-1β) and tumor necrosis factor-α (TNF-α) levels in vitro and in vivo. Furthermore, NP1 up-regulated the expression of miR-6328, which, in turn, down-regulated kappa B kinase β (IKKβ). IKKβ reduced the phosphorylation of nuclear factor-kappa B p65 (NF-κB p65) and inhibitor of NF-κB (I-κB), thereby inhibiting activation of the NF-κB pathway.

**Conclusions:**

The newly discovered non-toxic peptide NP1 (‘FLPAAICLVIKTC’) exerted neuroprotective effects on cerebral ischemia–reperfusion injury by reducing inflammation via the miR-6328/IKKβ/NF-κB axis. Our findings not only provide an exogenous peptide drug candidate and endogenous small nucleic acid drug candidate but also a new drug target for the treatment of IS. This study highlights the importance of peptides in the development of new drugs, elucidation of pathological mechanisms, and discovery of new drug targets.

## Introduction

Cerebral stroke is an acute cerebrovascular disease that includes hemorrhagic and ischemic stroke (IS). IS, which accounts for ~ 70% of all strokes, is caused by stenosis or occlusion of arteries supplying blood and oxygen to the brain [1, 2]. Stroke is one of the leading causes of death globally [3, 4]. Hypoxia and cerebral ischemia can lead to severe clinical symptoms, such as hemiplegia, hemianopia, aphasia, sensory disturbance, and decrease in consciousness [5]. These symptoms are caused by a series of complex pathological changes, including brain oxidative stress, neuroexcitotoxicity, and neuroinflammation [6]. Inflammation can have a considerable impact on prognosis after cerebral ischemia reperfusion [7]. With the increase in reactive oxygen species (ROS) and microglial activation in cerebrovascular disease, adhesion molecules are induced and enter the microvessels, leading to occlusion [8]. Inflammatory cells also release a variety of toxic substances, such as matrix metalloproteinases and nitric oxide, which can exacerbate neuronal death and disrupt the extracellular matrix and blood–brain barrier [9], leading to death or severe and lasting neurological damage [10]. Therefore, inhibition of inflammation is crucial for the effective treatment of IS.

The occurrence of inflammation is affected by many molecules and signaling pathways, including mitogen-activated protein kinase (MAPK), nuclear transcription factor-κB (NF-κB), and phosphatidylinositol 3-kinase/protein kinase B (PI3K/Akt) [11]. Among them, NF-κB is a key nuclear transcription factor, and activation of the NF-κB pathway is closely related to the occurrence of neuroinflammation [12]. Ischemia–reperfusion (I/R) injury activates the NF-κB pathway, which releases NF-κB from the cytoplasmic NF-κB/I-κB complex to undergo nuclear translocation, thereby initiating the transcription of target genes, including interleukin-1β (IL-1β) and tumor necrosis factor-α (TNF-α) [13]. Thus, the NF-κB signaling pathway is an important target for studying neuroinflammation in cerebral I/R [14].

Regulation of the NF-κB signaling pathway is affected by many factors. In recent years, the regulation of microRNAs (miRNAs) involved in the NF-κB signaling pathway has attracted increasing attention. MiRNAs are a class of non-coding RNAs consisting of 20–25 nucleotides, which can inhibit the translation of mRNAs by binding to their 3′ untranslated region (UTR) [15]. MiRNAs regulate various pathophysiological processes in the development of IS, such as neuronal death, oxidative stress, and inflammation [16]. For example, miR-21 reduces neuronal apoptosis during cerebral ischemia by targeting the Fas ligand, miR-148a-3p modulates Rock2 expression to alter oxygen–glucose deprivation/reperfusion (OGD/R)-induced oxidative stress [17], and miR-421-3p inhibits activation of the NF-κB pathway and reduces inflammation after cerebral ischemia [18]. Thus, miRNAs may be important biomarkers for IS prognosis [19–22] and the discovery of novel miRNAs may provide new therapeutic targets for IS therapy and prognosis [23].

The high rates of IS-related disability and mortality place a severe burden on patients, their families, and society. However, effective treatments and drugs are currently lacking [24]. Recombinant tissue plasminogen activator (rtPA) thrombolysis remains the only treatment approved by the US Food and Drug Administration (FDA). While rtPA thrombolysis can break down blood clots, it cannot rescue neuronal damage caused by hypoxia and must be administered within a demanding time window (4.5 h) [25]. Furthermore, reperfusion after thrombolysis can cause severe secondary damage to brain tissue and the cerebrovascular system, aggravating the inflammatory response and risk of hemorrhagic transformation (HT), disability, and mortality [26]. Thus, the discovery of alternative drugs and new drug targets for cerebral I/R injury is critical.

In recent years, neuroprotective agents derived from plants such as scutellarin and gastrodin have been reported [27, 28]. However, these are far from enough for the needs of the disease [29]. Animal-derived active peptides have attracted growing attention due to their natural origin, short structure, strong activity, high safety, and low toxicity [30, 31]. Active peptides Hi1a from Australian funnel-web spider *Hadronyche infensa* and exendin-4 from *Heloderma suspectum* venom have been found to have an anti-stroke activity, effectively alleviating the recovery of non-penumbra after stroke and improving prognosis [32, 33]. Despite this, research on the treatment of ischemic encephalopathy with peptide drugs remains poor, and the inhibition of inflammation and neuroprotective effects of peptides need further exploration [34]. Amphibians are a natural treasure trove of bioactive peptides, with their skin and skin secretions containing structurally rich bioactive peptides, with antibacterial, antioxidant, and anti-inflammatory activities [30, 35]. We previously demonstrated that a frog-derived peptide (OM-LV20) attenuated cerebral ischemia/reperfusion injury, exerted neuroprotective effects, and promoted spinal cord recovery in rats [24, 36].

In the current research, we identified a new non-toxic peptide (NP1, amino acid sequence ‘FLPAAICLVIKTC’), which showed strong neuroprotective effects against cerebral I/R injury by reducing inflammation via the miR-6328/IKKβ/NF-κB axis. Our results not only provide an exogenous peptide drug candidate and endogenous small nucleic acid drug candidate but also a new drug target for the treatment of IS. Our research highlights the importance of peptides in the creation of new drugs, elucidation of pathological mechanisms, and discovery of new drug targets.

## Materials and methods

### Screening of cDNA-encoding peptide from *Rana limnocharis* skin

The cDNA-encoding peptide NP1 was screened and obtained as per our previous studies [30, 37]. In brief, the skin of individual *R. limnocharis* was rinsed with purified water. After being killed, the back skin of *R. limnocharis* was quickly stripped, cut into pieces with scissors and transferred to liquid nitrogen for grinding to powder. mRNA was extracted from total RNA using the Absolutely mRNA Purification Kit (Stratagene, Canada). After that, mRNA was used as a template, first and second strand cDNAs were synthesized using the SMART cDNA Library Construction Kit (Clontech, Canada). Using the second strand cDNA as a template, cDNA encoding mature NP1 was screened, polymerase chain reaction (PCR) primers were 5′PCR primers (5′-CCAAA (G/C) ATGTTCACC (T/A) TGAAGAA-3′) and 3′PCR primers (5′ATTCAGGCCGAGGCC GACA TG-3′). The obtained PCR products were cloned into *Escherichia coli* DH5a active cells and DNA sequenced using an Applied Biosystems DNA sequencer (ABI 3730XL, USA). Finally, the cDNA sequence encoding NP1 was obtained.

### Synthesis of peptide and prediction of advanced structure

The NP1 (‘FLPAAICLVIKTC’) and FITC-labeled NP1 peptides (purity > 95%) were solid-phase synthesized by Wuhan Tiande Biotechnology Co., Ltd. (Wuhan, China). The chemical structure of NP1 was drawn using ChemDraw^®^ and the de novo structure of the peptide was predicted using the PEP-FOLD3 online service in accordance with our previous report [30].

### Animal care and use

Sprague–Dawley (SD) rats (male and female; 8 weeks of age; 280–300 g) were purchased from Hunan Slack Jingda Experimental Animal Co., Ltd. (Hunan, China). Males were used for the middle cerebral artery occlusion/reperfusion (MCAO/R) model. Males and females were used for the acute toxicity experiments. All animal care and experimental procedures followed the Provision and General Recommendation of the Chinese Experimental Animals Administration Legislation. Rats were housed at 22 ± 2 °C under a 12 h light/dark cycle with ad libitum water and food. All experimental treatments were carried out in compliance with the requirements of the Ethics Committee of Kunming Medical University (KMMU2021271).

### Hemolytic activity and acute toxicity assays

The hemolytic activity of NP1 (0.1, 1, 10, and 100 nM) was detected according to previously reported methods [38]. Venous blood was collected from the SD rats, then washed with normal saline three times and centrifuged at 2500×*g* for 5 min at room temperature. The resulting supernatant was removed to obtain fresh red blood cells, which were diluted with physiological saline to obtain a 3% red blood cell solution. Different concentrations of NP1 (0.1, 1, 10, and 100 nM) were added to the solution at the ratio of 1:1 (V/V), respectively. Physiological saline was used as the negative control and 0.1% Triton X-100 was used as the positive control. The samples were incubated at 37 °C for 30 min and centrifuged at 2500×*g* for 5 min at room temperature, with the supernatant used to measure optical density (OD) at 540 nm. Hemolytic activity (%) was expressed as [(*A*_sample_) × 100]/*A*_Triton X-100_. Rats were intraperitoneally injected with NP1 (0.1, 1, or 10 nmol/kg) or normal saline (control group). Rat mortality was observed and recorded over one week to detect acute toxicity of NP1. In addition, rat tissue samples (cortex, hippocampus, liver, heart, spleen, lung, and kidney) were quickly removed following perfusion with 4% paraformaldehyde. All tissues were fixed, embedded in paraffin, and stained with hematoxylin and eosin (H&E) according to previous methods [39], and all sections were viewed to further determine acute toxicity of the different concentrations of NP1.

### Detection of NP1 crossing blood–brain barrier

The ability of NP1 to cross the blood–brain barrier was verified according to previously reported methods [40]. After the rats were anesthetized, the neck was incised down the middle and gland tissue and fascia of the neck were bluntly separated. The common carotid artery, external carotid artery, and internal carotid artery were exposed and separated. The proximal end of the common carotid artery and distal end of the internal carotid artery were clamped with vascular clips. The external carotid artery was ligated, then cut at the distal end so that the external and internal carotid arteries presented a 180° angle. A needle was inserted into the external carotid artery and fixed, with the vascular clamp holding the common carotid artery then released, followed by an injection of NP1 with FITC (dose: 10 nmol/kg). After injection, the common carotid artery was clamped, and the needle was slowly removed. The external carotid artery incision was ligated, and the vascular clip was removed to restore blood flow of the cerebral artery. Finally, the subcutaneous tissue and skin were sutured. At 1 h after the NP1 injection, the rats were deeply anesthetized and fully perfused, after which their brains were extracted, removed from light, and immersed in 4% paraformaldehyde. The preserved brain tissues were sent to Kunming Pulis Technology Co., Ltd. (Kunming, China) for fluorescence photography.

### Evaluation of neuroprotective effects of NP1 in rats

The MCAO/R model was established in rats to simulate I/R injury and the neuroprotective effects of NP1 were evaluated according to previous methods [41]. After the rats were anesthetized, the neck was incised down the middle and the gland tissue and fascia of the neck were bluntly separated. The common carotid artery, external carotid artery, and internal carotid artery were exposed and separated. The proximal end of the common carotid artery and distal end of the internal carotid artery were clamped with vascular clips. The external carotid artery was ligated and cut at the distal end so that the external and internal carotid arteries presented a 180-degree angle. A nylon suture (Cinontech, Beijing, China) was slowly insert from the incision to the internal carotid artery until a slight resistance was felt, i.e., the suture reached the middle cerebral artery. The suture was placed at the middle cerebral artery for 2 h, then slowly removed. The external carotid artery incision was ligated, and the vascular clip was removed to restore blood flow of the middle cerebral artery. Finally, the subcutaneous tissue and skin were sutured.

The rats were randomized into five groups: i.e., sham, I/R, and NP1 treatment (I/R + NP1 0.1 nmol/kg; I/R + NP1 1 nmol/kg; I/R + NP1 10 nmol/kg) groups. The NP1 treatment groups received an intraperitoneal injection of NP1 at three different concentrations, respectively, with the first injection given immediately after I/R operation. Doses were then administered every 12 h for three consecutive days.

The rats in each group were over-anesthetized and quickly decapitated at various time points (24 h, 48 h and 72 h) after I/R, depending on the purpose of the experiment. 2,3,5-Triphenyltetrazolium chloride (TTC) staining was performed to evaluate the effects of NP1 on cerebral infarct volume in each group of rats after MCAO/R according to previous research [42]. Total area and infarct area were calculated using ImageJ v1.8.0, expressed as (infarct volume/whole brain volume) × 100%.

Rats with a Longa score of 0 or 4 were excluded, and the remaining rats were evaluated using the modified Neurological Severity Score (mNSS) system to clarify the effects of NP1 on the degree of neurological deficit in rats after MCAO/R. Three people performed scoring using the single-blind method [43]. All scoring results were statistically aggregated for analysis.

### Effects of NP1 on brain cell injury and neuronal survival in rats after I/R

At 72 h after I/R, rat brains were quickly removed following perfusion with 4% paraformaldehyde. The brain tissue was fixed, embedded in paraffin, and stained with hematoxylin and eosin (H&E) or Nissl according to previous methods [39]. All sections were viewed using a Primovert microscope (Zeiss, Germany) to analyze changes in cell morphology and state. Viable neuron number was quantified using ImageJ software.

### Effects of NP1 on protein levels of IL-1β, TNF-α, IKKβ, NF-κB p65, I-κB, p-NF-κB p65, and p-I-κB in brain tissue and PC12 cells

Proteins were extracted from brain tissue at different time periods (24 h, 48 h and 72 h) after I/R and from PC12 cells 24 h after OGD/R. Protein concentration was determined and western blotting was performed according to previous methods [44]. The primary antibodies used included IL-1β (1:2000), TNF-α (1:1000), IKKβ (1:1 000), NF-κB p65 (1:2000), I-κB (1:2000) (Proteintech, Hubei, China), p-NF-κB p65 (1:1000), and p-I-κB (1:1000) (Cell Signaling Technology, MA, USA). Horseradish peroxidase (HRP)-conjugated secondary antibodies (1:10,000; (Proteintech, Hubei, China)) were also used. Band signals were visualized and exposed using an enhanced chemiluminescence kit (Biosharp Life Science, Anhui, China). Results were assessed using ImageJ software.

### Evaluation of neuroprotective effects of NP1 in PC12 cells

The PC12 cells were provided by the Cell Bank of the Kunming Institute of Zoology, Chinese Academy of Sciences. The cells were randomized into five groups: i.e., Control, OGD/R, and OGD/R + NP1 (0.1, 1, and 10 nM) groups. All cells were cultured as per previous study [45].

An OGD/R-induced cellular model was constructed to evaluate the neuroprotective effects of NP1 in PC12 cells. The well-adhered PC12 cells were washed with phosphate-buffered saline (PBS, pH 7.2) three times, with glucose-free Dulbecco’s Modified Eagle Medium (DMEM) then added. The OGD-induced cells were cultured in a three-gas incubator containing 95% N_2_ and 5% CO_2_ for 4 h, followed by the addition of normal DMEM/F12 at 37 °C with 5% CO_2_ for 24 h.

The effects of different concentrations of NP1 on cell viability after OGD/R were evaluated by MTS assay. Cells were seeded in 96-well plates at a density of 4000 cells/well. After cell adherence, the cell groups (except the control) were subjected to OGD induction as described above. The NP1-treated groups were administered different concentrations of NP1 (0.1, 1, and 10 nM) immediately after OGD induction and further cultured for 24 h after re-oxygenation. Finally, 20 μL of MTS was added to each well, followed by incubation at 37 °C in the dark for 3 h. The optical density (OD) of each well at 490 nm was detected using a microplate reader.

### MiRNA sequencing

The cerebral cortical tissues of rats in the sham, I/R, and most effective NP1 dose (10 nmol/kg) groups were quickly removed, stored in liquid nitrogen, and sent to Beijing Guoke Biotechnology Co., Ltd. (China) for miRNA sequencing analysis. The raw data were subjected to Gene Ontology (‘GO: TermFinder’ (http://www.geneontology.org/)) and Kyoto Encyclopedia of Genes and Genomes (KEGG) pathway analysis (http://www.genome.jp/KEGG/). The miRNA target genes were also predicted and analyzed [30].

### Effects of NP1 on mRNA levels of IKKβ and expression of miR-6328 in brain tissue and PC12 cells

MiRNA and mRNA were extracted from rat brain tissues after different time periods (24 h, 48 h and 72 h) of I/R and from PC12 cells after 24 h of OGD/R using an RNA extraction kit (GeneCopoeia, Guangzhou, China). The miRNAs and mRNAs were reverse-transcribed and single-stranded cDNA was synthesized using a Prime Script Reagent Kit (GeneCopoeia, China). Quantitative real-time polymerase chain reaction (qRT-PCR) using specific primers miR-6328 (forward, CTCTGAGCCCCCGCAAA), U6 (HsnRNA U6 primer, GeneCopoeia, Rockville, MD, USA), IKKβ (forward, GTGGTGAGGCTCATGAACGA; reverse, CGGAAGCGGCACAGGATGACC), IL-1β (forward, GGGATGATGACGACCTGCTA; reverse, CCACTTGTTGGCTTATGTTCTG), TNF-α (forward, AGAACTCCAGGCGGTGTCT; reverse, GAGCCCATTTGGGAACTTCT) and Actin (forward, CAGCCTTCCTTCCTGGGTATG; reverse, TAGAGCCACCAATCCACACAG) was performed using a PCR instrument (Life Technologies, Thermo, USA). The 2^−ΔΔct^ method was used to calculate relative expression in samples and control [46].

### Effects of miR-6328 on IKKβ expression in PC12 cells

To clarify the regulatory relationship and mode between miR-6328 and IKKβ, cells were transfected with 100 nM miR-6328 inhibitor (Ribobio, Guangzhou, China) and its corresponding negative control (NCi) (Ribobio, Guangzhou, China) or 50 nM miR-6328 mimic (Ribobio, Guangzhou, China) and its negative control (NCm) (Ribobio, Guangzhou, China) using transfection reagent (Ribobio, Guangzhou, China) for 24 h according to the manufacturer’s requirements. Cells were then collected for subsequent experiments [47, 48].

Dual-luciferase reporter assays were carried out by Platzer Biotechnology Co., Ltd. according to previous methods [49].

### Statistical analysis

Pairwise comparisons were conducted by one-way analysis of variance (ANOVA) with the Bonferroni post-hoc test. Comparisons between two groups were carried out with the Student two-tailed *t*-test using GraphPad Prism v8.0 (La Jolla, CA, USA). All data were expressed as means ± standard error of the mean (SEM) and *P* < 0.05 was considered statistically significant [42].

## Results

### Discovery and molecular properties of novel peptide NP1

**Fig. 1:**
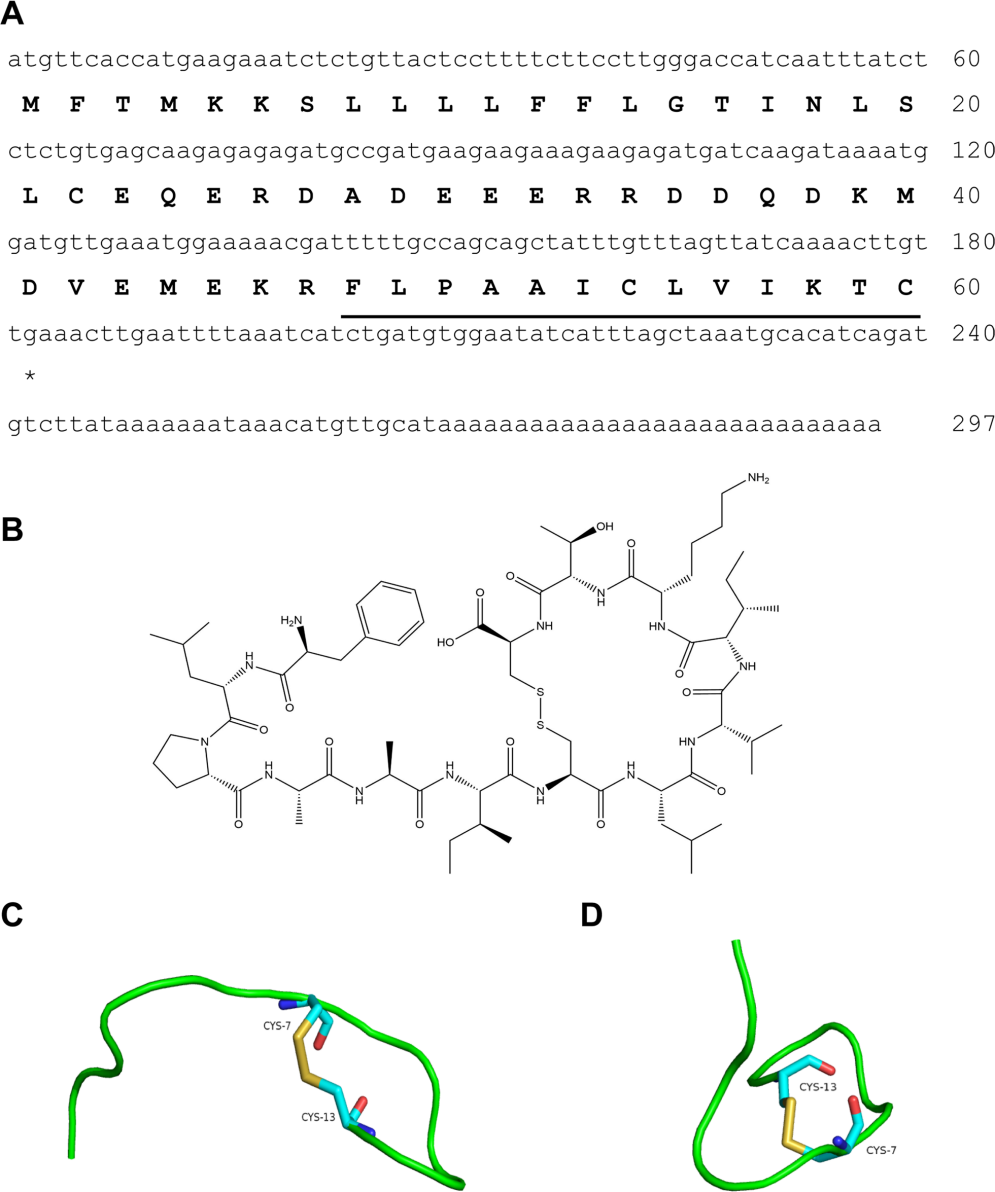
Molecular properties of novel peptide NP1. **A** Prepropeptide NP1 encoded by a 297-bp cDNA sequence and consisting of 60 amino acid residues. Mature peptide sequence is underlined. **B** Chemical structure of NP1. **C**, **D** Advanced structure of NP1 observed from different angles. Red: oxygen atoms; yellow: sulfur atoms; blue: nitrogen atoms; blue-green represent higher-order structures formed

A peptide precursor consisting of 60 amino acid residues encoded by a 297-bp cDNA sequence was obtained from the skin of *R. limnocharis*. The overall structure of the NP1 precursor was similar to that of other frog peptides, and included signal peptides, acidic fragments, enzyme cleavage site KR, and mature peptides. The mature peptide sequence was predicted to be ‘FLPAAICLVIKTC’ (Fig. 1A), different from all other bioactive frog peptides reported in the NCBI database. Thus, it was considered a new sequence and named Neuroprotective Peptide 1 (NP1) (molecular weight: 1 389.76 Da). The chemical structure of NP1 is shown in Fig. 1B. The advanced structure of NP1 predicted by PEP-FOLD3 is shown in Fig. 1C, D, which indicated that the peptide contained a pair of intramolecular disulfide bonds between C^7^ and C^13^, suggesting a certain degree of stability.

### NP1 exhibited no hemolytic activity against rat erythrocytes and no acute toxicity

**Fig. 2:**
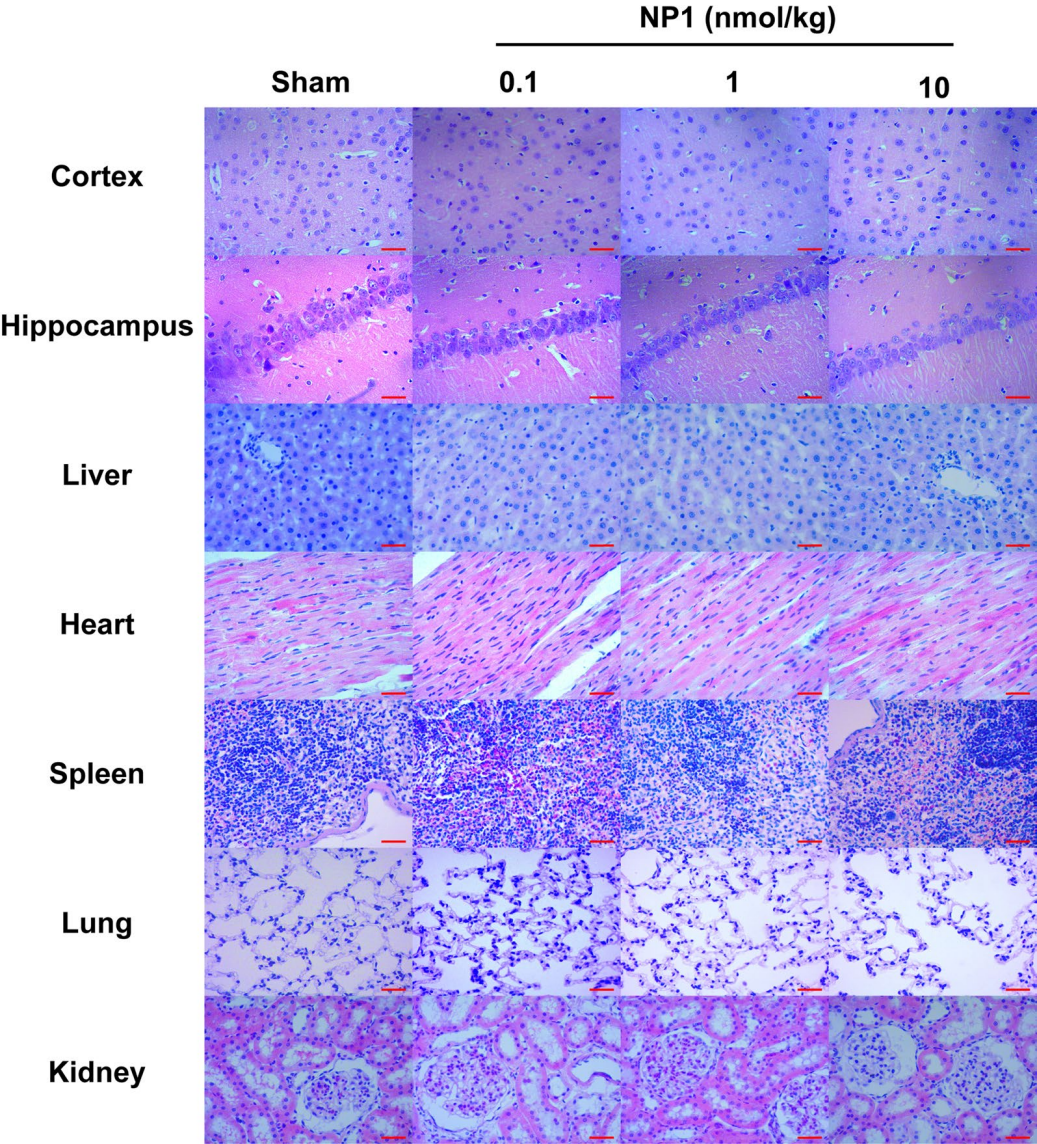
NP1 had no toxicity to the main organs of rats (*n* = 3). Scale is 75 μm

Compared with the saline group, the hemolysis rate of NP1 (0.1, 1, 10, and 100 nM) against rat erythrocytes was not significantly different (Table 1), indicating that the peptide exhibited no hemolytic activity in vitro. As shown in Table 2, to test the safety of NP1 in vivo, female and male rats were intraperitoneally injected with different concentrations of NP1 (0.1, 1, 10 nmol/kg). However, no abnormal phenomena or deaths were observed after one week of continuous observation. Compared with the saline group, H&E staining of the main rat organs (cortex, hippocampus, liver, heart, spleen, lung, and kidney) in the different NP1 concentration groups showed no obvious pathological changes (Fig. 2), indicating that NP1 was not acutely toxic. These results suggest that the novel peptide is safe, laying the foundation for subsequent experiments.

**Table 1:** Effects of NP1 on hemolytic activity in rat erythrocytes

Group	Hemolysis ratio
0.1% Triton X-100 Saline NP1 100 pM NP1 1 nM NP1 10 nM NP1 100 nM	100 6.6 ± 0.11 5.7 ± 0.10 6.2 ± 0.09 6.0 ± 0.05 5.3 ± 0.10

Data are means ± SD (*n* = 6)

**Table 2:** Acute toxicity of NP1 in rats

Group and dose	Male (*n*)	Female (*n*)	Mortality
Saline NP1 0.1 nmol/kg NP1 1 nmol/kg NP1 10 nmol/kg	3 3 3 3	3 3 3 3	0 0 0 0
(*n* = 3)

### Ability of NP1 to cross blood–brain barrier

**Fig. 3:**
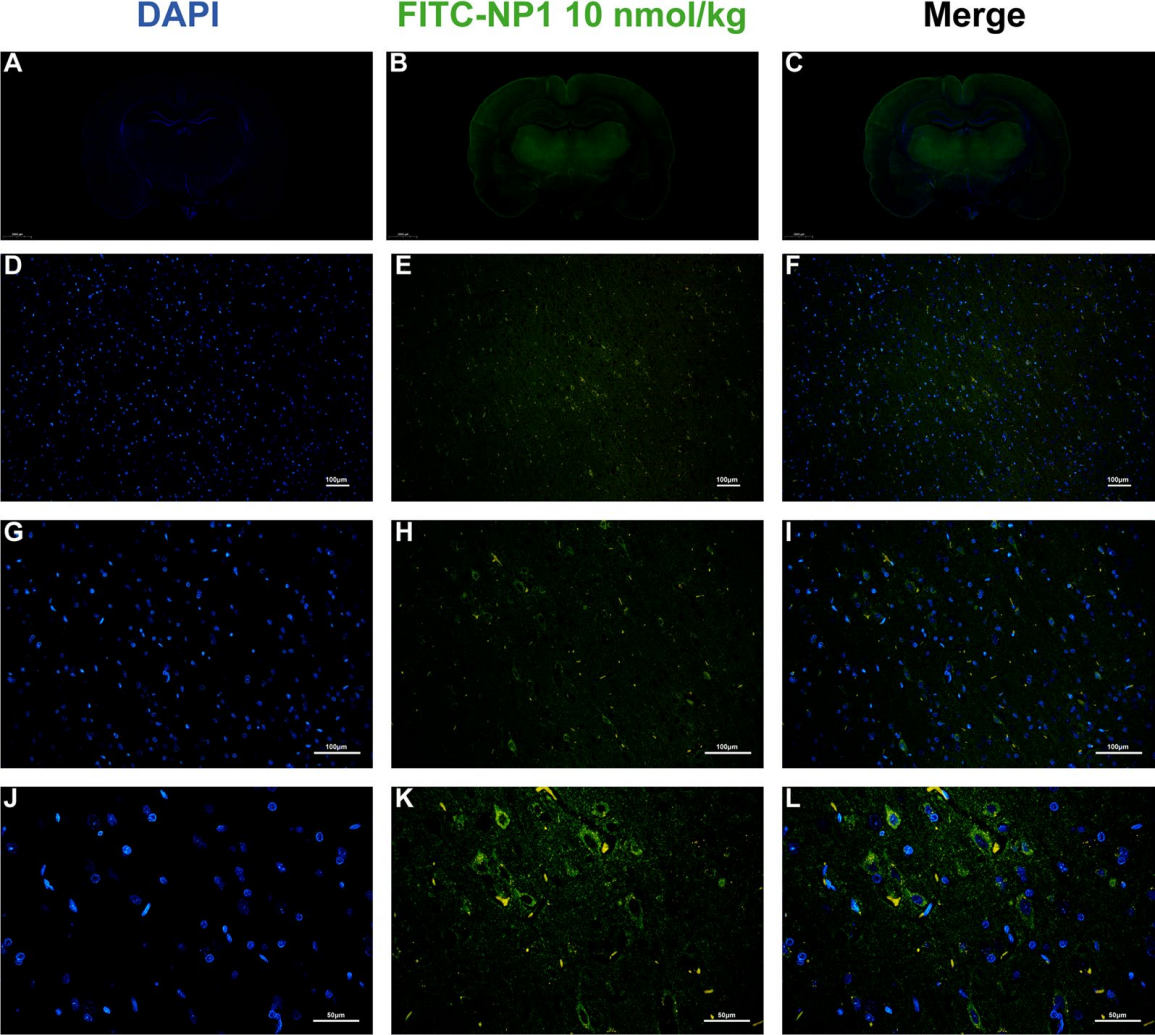
NP1 effectively crossed the blood–brain barrier (*n* = 3). **A**–**C** Fluorescence scan of whole brain tissue of rats at 1 h after FITC-NP1 injection. **D**, **G**, **J** DAPI staining images of rat brain tissue sections at different magnifications at 1 h after FITC-NP1 injection. **E**, **H**, **K** FITC fluorescence signal diagrams of brain tissue slices under different magnifications at 1 h after FITC-NP1 injection. **F**, **I**, **L** Merged images of rat brain tissue sections at different magnification at 1 h after FITC-NP1 injection

As shown in Fig. 3, 1 h after injection, the fluorescence signal of FITC-labeled NP1 appeared around the blood vessels and spread to the surrounding brain tissues, with the cortex, hippocampus, and thalamus showing the most obvious signals (Fig. 3A–C). After magnification, the fluorescence signal appeared in the cytoplasm (Fig. 3D–L). These results indicated that NP1 crossed the blood–brain barrier and entered the cytoplasm.

### NP1 reduced cerebral infarct volume and ameliorated neurological deficits in rats after I/R

**Fig. 4:**
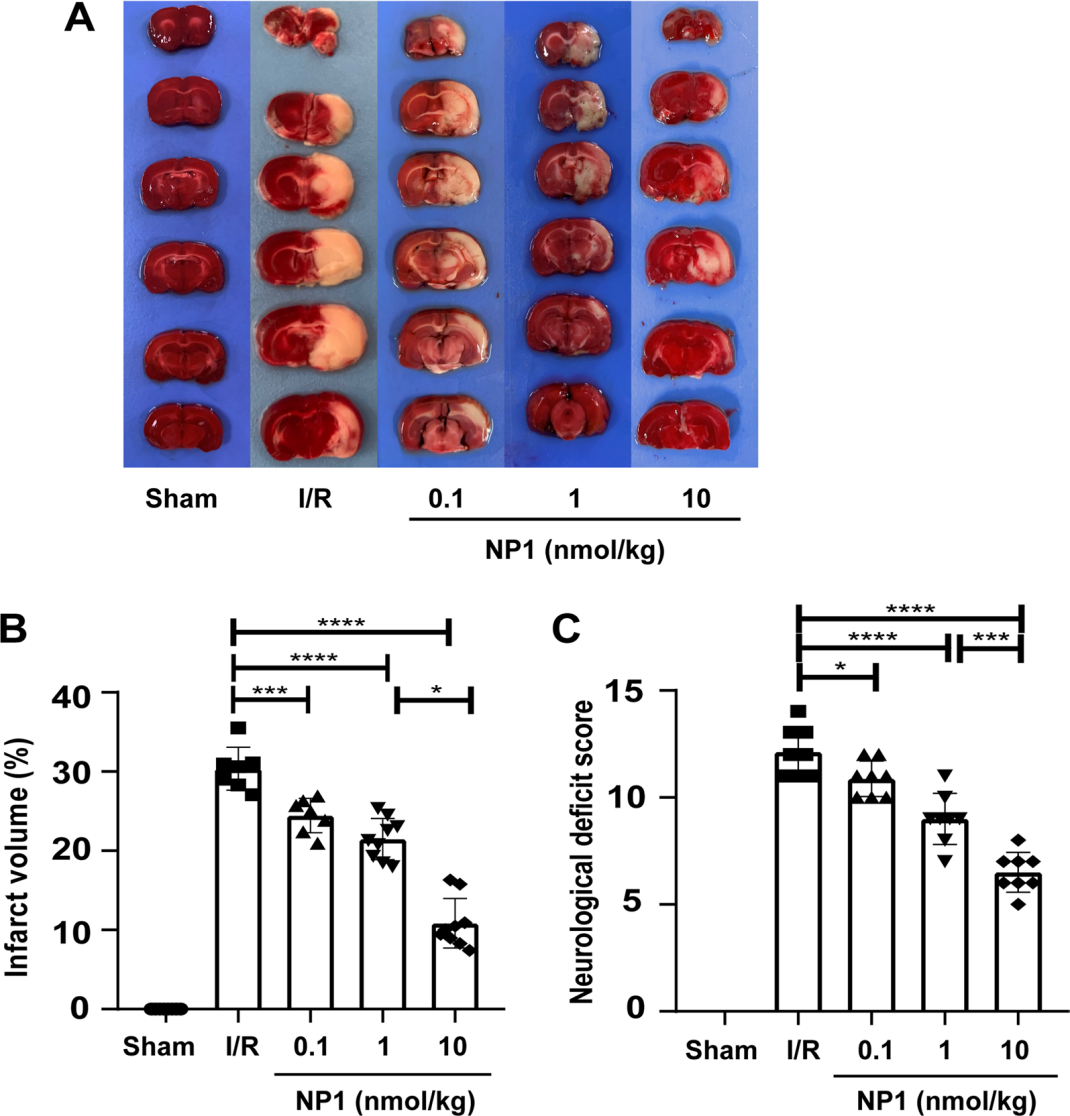
NP1 reduced cerebral infarct volume and ameliorated neurological dysfunction after I/R. **A** Most symbolic images of rat brain tissue in each group after TTC staining. **B** Quantification of cerebral infarct area in each group of rats. Eight rats in sham group, seven rats in I/R and 0.1 nmol/kg groups, respectively, nine rats in 1 and 10 nmol/kg groups, respectively. **C** Quantification of mNSS in each group (*n* = 8). Data are presented as means ± SEM. *P < 0.05, ***P < 0.001, ****P < 0.0001

Symptoms of stroke developed after MCAO/R in rats. TTC staining was applied to detect cerebral infarct size in rats after I/R. Results showed that I/R injury caused a large area of brain infarction compared to the sham group. After NP1 treatment, especially at 10 nmol/kg, cerebral infarct volume was reduced and infarct conditions in the penumbra were significantly improved (*P* < 0.0001; Fig. 4A, B). Furthermore, the mNSS results showed that the I/R group had severe neurological deficits compared to the sham group. However, these neurological deficits were significantly improved after intraperitoneal administration of NP1, especially at the 10 nmol/kg dose (*P* < 0.0001; Fig. 4C).

### NP1 ameliorated cell damage and improved neuronal survival in rat brains after I/R

**Fig. 5:**
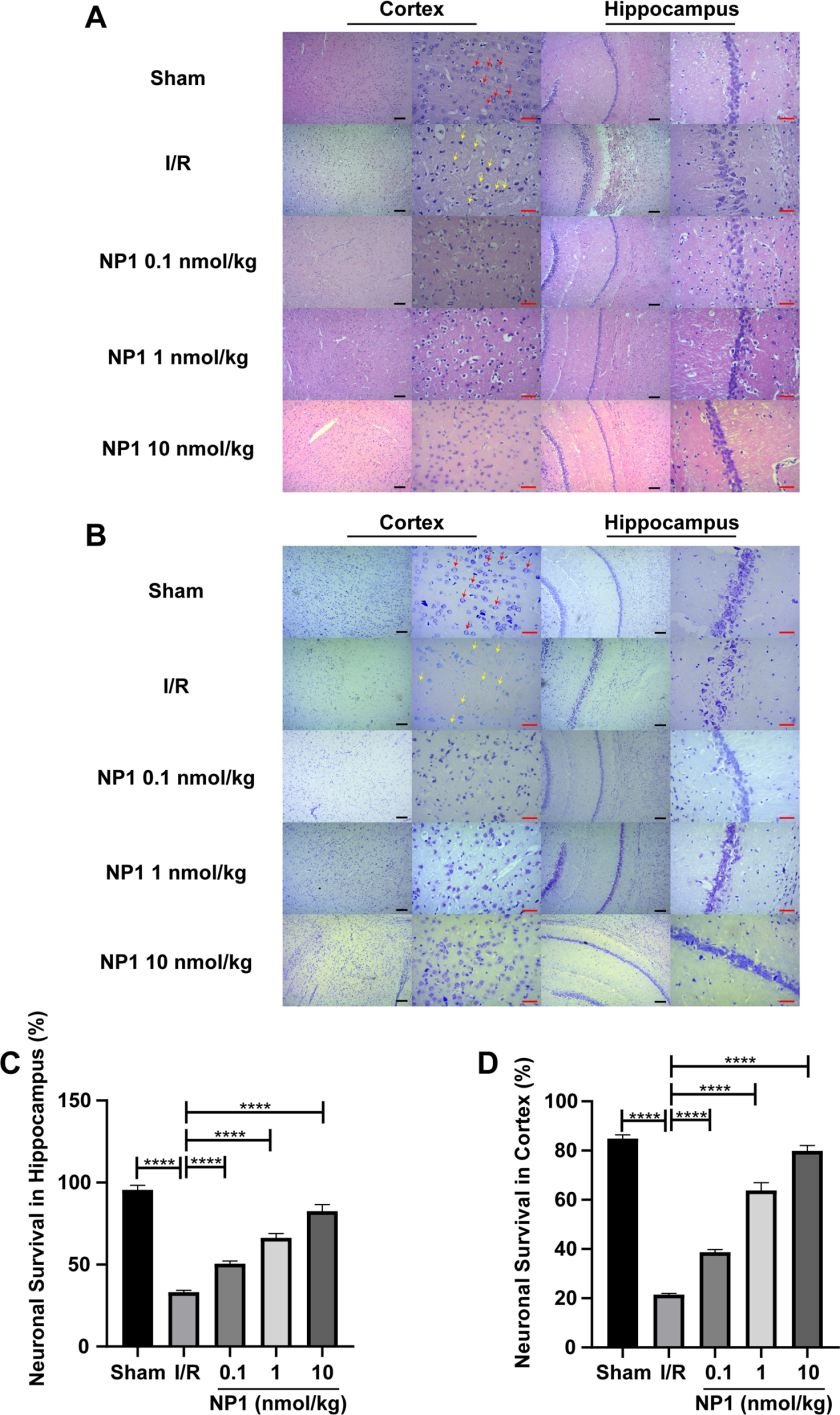
NP1 reduced damage to brain cells and increased neuronal survival (*n* = 3). **A** H&E staining of cerebral cortex and hippocampus of rats. **B** Nissl staining of cerebral cortex and hippocampus of rats. **C**, **D** Quantification of neuronal survival in Nissl-stained cortical and hippocampal areas. Black scale is 100 μm, red scale is 75 μm. Normal cells are indicated by red arrows, necrotic cells are indicated by yellow arrows. Data are presented as means ± SEM. ****P < 0.0001

Morphological changes in the cerebral cortex and hippocampus of rats were observed by H&E and Nissl staining. As shown in Fig. 5A, B, cortical and hippocampal cells in the sham group were normal, with lightly stained cytoplasm, clear nuclei, intact Nissl bodies, and dense and neatly arranged hippocampal cells. In the I/R group, the cortical and hippocampal cells were damaged and shrunken, with deeply stained cytoplasm, pyknotic or fragmented nuclei, and loosely arranged and disordered hippocampal cells. However, NP1 treatment effectively attenuated cerebral ischemia-hypoxia-induced cell damage and necrosis and improved hippocampal cell arrangement. Quantification of Nissl staining revealed a significant reduction in neuronal survival within the cortex and hippocampus after I/R (*P* < 0.0001). However, NP1 application significantly improved and protected neuronal survival (Fig. 5C, D), with the optimal effect achieved at 10 nmol/kg (*P* < 0.0001).

### NP1 reduced IL-1β and TNF-α expression in rat brains after I/R

**Fig. 6:**
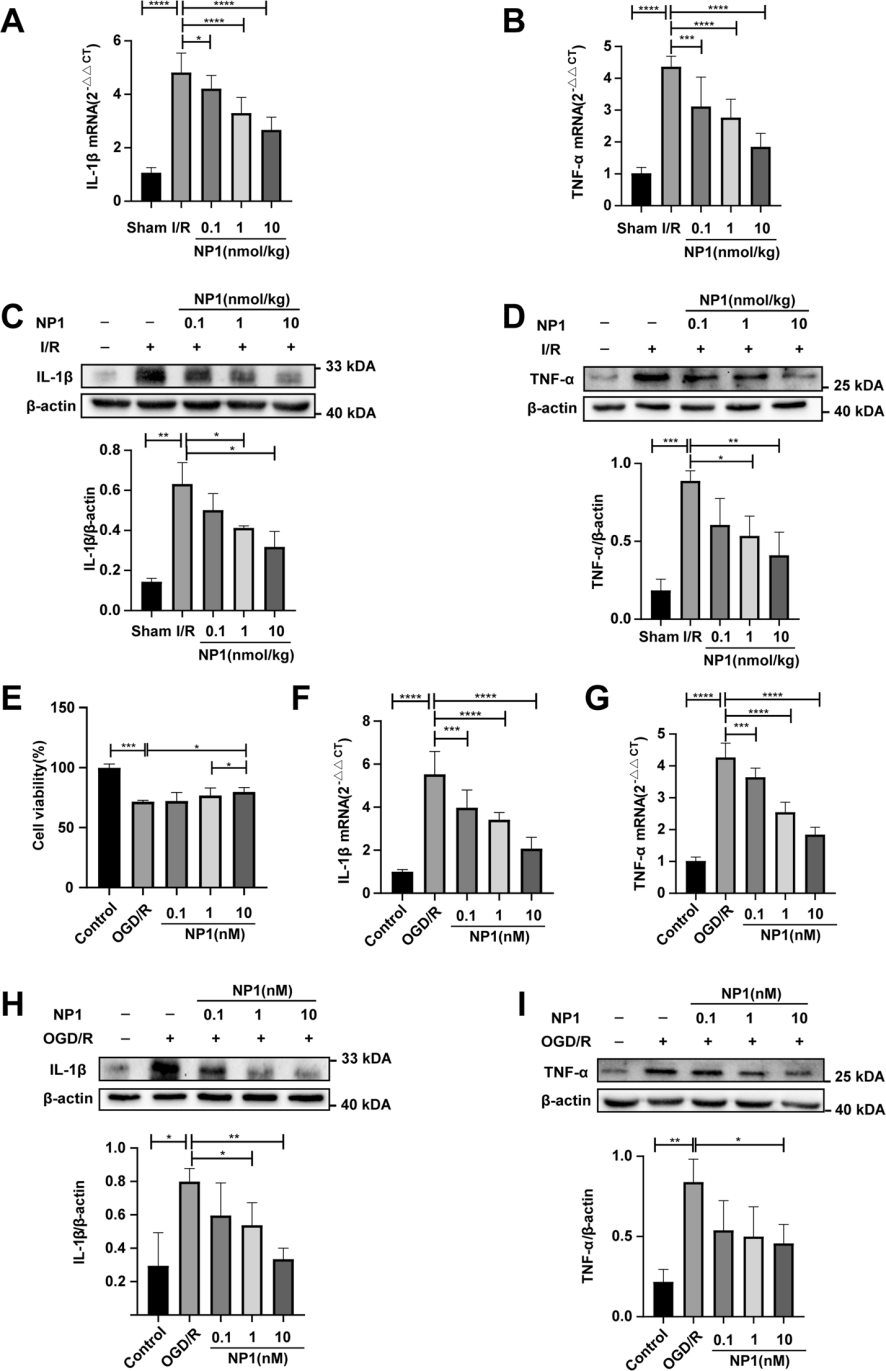
NP1 reduced IL-1β and TNF-α content and improved cell survival. **A** NP1 reduced overexpression of IL-1β at the mRNA level after I/R injury in rats (*n* = 3). **B** NP1 reduced overexpression of TNF-α at the mRNA level after I/R injury in rats (*n* = 3). **C** NP1 reduced overexpression of IL-1β at the protein level after I/R injury in rats, with expression levels in each group shown (*n* = 3). **D** NP1 reduced overexpression of TNF-α at the protein level after I/R injury in rats, with expression levels in each group shown (*n* = 3). **E** NP1 reduced cell death caused by OGD/R in MTS cell viability assay. **F** NP1 reduced overexpression of IL-1β at the mRNA level after OGD/R injury in PC12 cells. **G** NP1 reduced overexpression of TNF-α at the mRNA level after OGD/R injury in PC12 cells. **H** NP1 reduced overexpression of IL-1β at the protein level after OGD/R injury in PC12 cells, with expression levels in each group shown. **I** NP1 reduced overexpression of TNF-α at the protein level after OGD/R injury in PC12 cells, with expression levels in each group shown. Data are presented as means ± SEM. *P < 0.05, **P < 0.01, ***P < 0.001, ****P < 0.0001

The occurrence of inflammation is important in the progression of IS. Excessive release and expression of inflammatory factors aggravate the inflammatory response and apoptosis, causing more serious damage to brain tissue. Here, the expression levels of IL-1β and TNF-α in the rat brain were detected by western blotting. As shown in Fig. 6A–D the mRNA and protein expression levels of IL-1β and TNF-α in the brain were significantly increased 72 h after I/R compared with the sham group. However, NP1 reduced the release of these factors, and the inhibitory effect on IL-1β and TNF-α expression was most pronounced at the 10 nmol/kg dose.

### NP1 enhanced PC12 cell viability and reduced IL-1β and TNF-α expression after OGD/R

To verify the neuroprotective properties of NP1 at the cellular level, PC12 cells were cultured to construct an OGD/R model, and the effects of NP1 on cell viability after OGD/R injury were determined by MTS assay. After OGD/R induction, PC12 cell death increased significantly, but was ameliorated by NP1 (10 nM) treatment (Fig. 6E). Furthermore, the cellular expression of IL-1β and TNF-α in each group were detected. Results indicated that OGD/R significantly increased IL-1β and TNF-α content in the PC12 cells compared with the control group. However, NP1 treatment (1 and 10 nM) inhibited the OGD/R-induced increases in IL-1β and TNF-α expression (Fig. 6F–I).

### Analysis of miRNA sequencing

**Fig. 7:**
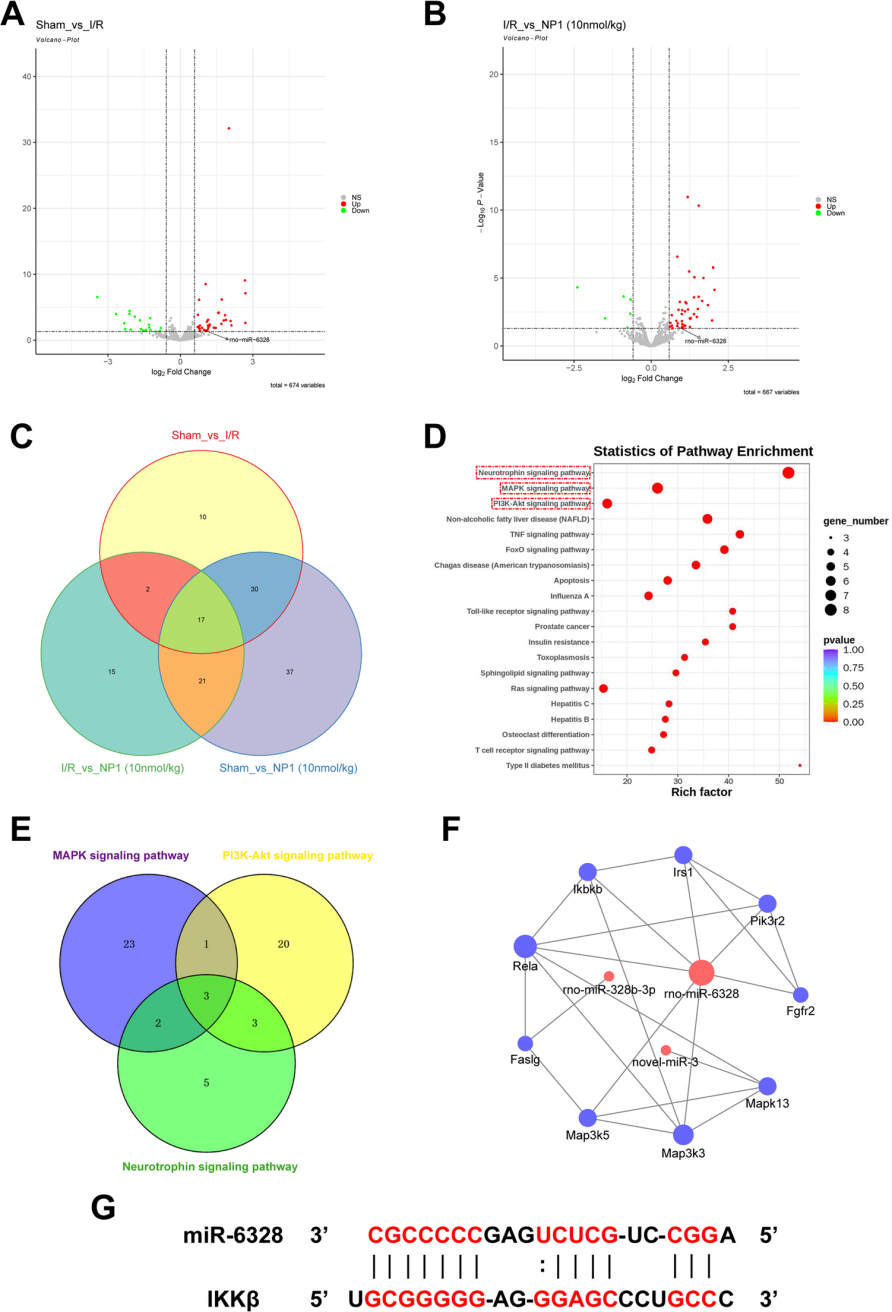
MiRNA sequencing analysis of cerebral cortex tissue in rats (*n* = 3). **A** In total, 59 miRNAs showed significant differential expression between the sham and I/R groups, with down-regulated miRNAs in green and up-regulated miRNAs in red. **B** In total, 55 miRNAs showed significant differential expression between the I/R and 10 nmol/kg NP1 groups, with down-regulated miRNAs in green and up-regulated miRNAs in red. **C** Venn diagram of number of differential miRNAs obtained after pairwise comparison of three groups, overlap is number of miRNAs simultaneously present in two or three groups. **D** Top 10 most significant visualizations after KEGG enrichment analysis of target genes of 19 differentially expressed miRNAs after intersection. **E** Venn diagram of number of target genes in MAPK, PI3K/Akt, and neurotrophin signaling pathways, overlap is number of target genes simultaneously present in two or three pathways. **F** Prediction of target proteins of significantly up-regulated miRNAs and their interactions. G. Prediction of binding site of miR-6328 to IKKβ

To explore the pathway by which NP1 exerts its neuroprotective effects, we performed miRNA sequencing analysis of the cerebral cortex in rats from the sham, I/R, and most effective NP1 dose (10 nmol/kg) groups. Results showed that 59 miRNAs exhibited significant differential expression in the sham group compared with the I/R group (Fig. 7A). Furthermore, 55 miRNAs exhibited significant differential expression in the 10 nmol/kg NP1 group compared with the I/R group (Fig. 7B). In total, 19 miRNAs showed significantly different expression in both comparison groups (Fig. 7C). KEGG pathway enrichment analysis of target genes of the 19 differentially expressed miRNAs was performed. The top 10 most significantly enriched pathways were selected for visualization, including the MAPK, PI3K/Akt, and neurotrophin signaling pathways (Fig. 7D). Further analysis found that IRS1, Faslg, PIK3R2, IKKβ, Rela, Fgfr2, MAP3K5, MAP3K3, and MAPK1 existed in two or three of these pathways. Interestingly, seven factors were predicted to be miR-6328-target genes (except Faslg regulated by rno-miR-328b-3p and MAPK13 regulated by novol-miR-3), and IKKβ co-existed in all three of the above pathways (Fig. 7E, F). Therefore, miR-6328 and IKKβ were further investigated, and their binding sites were predicted (Fig. 7G).

### Effects of NP1 on miR-6328 and IKKβ expression in rat brain after I/R and IKKβ expression at 24, 48, and 72 h after I/R

**Fig. 8:**
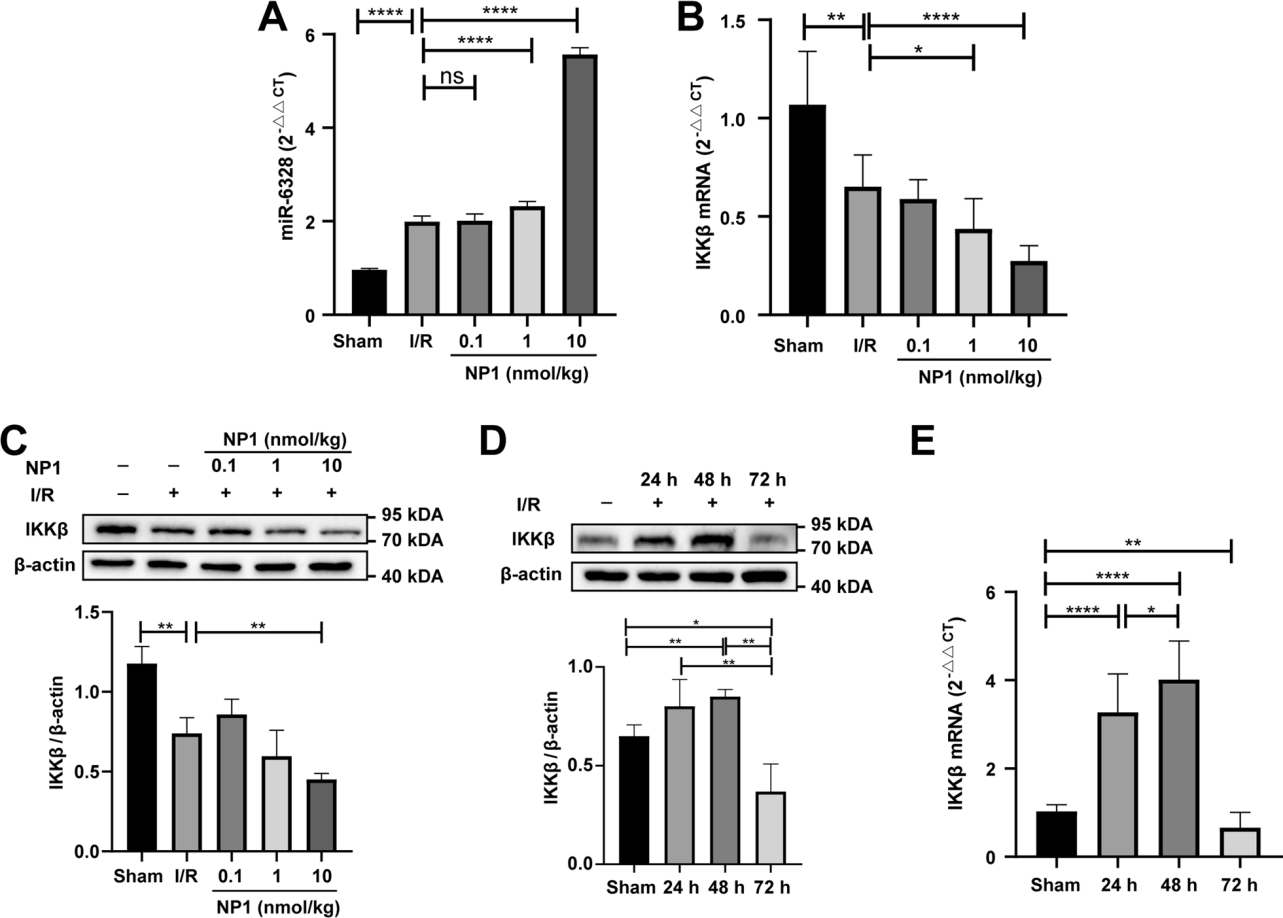
Validation of miRNA sequencing analysis (*n* = 3). **A** Content of miR-6328 in brain tissue of rats. **B** Content of IKKβ at the mRNA level in brain tissue of rats. **C** Content of IKKβ at the protein level in brain tissue of rats. **D** Content of IKKβ at the protein level in brain tissue of rats at 24, 48, and 72 h after I/R. **E** Content of IKKβ at the mRNA level in brain tissue of rats at 24, 48, and 72 h after I/R. Data are presented as means ± SEM. *P < 0.05, **P < 0.01, ****P < 0.0001

To verify the miRNA sequencing results, changes in the expression of miR-6328 and IKKβ after I/R injury were verified by qRT-PCR and western blot analysis. Results showed that miR-6328 expression increased after I/R, and this trend was more significant after NP1 treatment (*P* < 0.0001; Fig. 8A). Furthermore, IKKβ expression at the mRNA and protein levels decreased somewhat after I/R injury, and this trend was more significant after NP1 treatment, especially at 1 and 10 nmol/kg (Fig. 8B, C). These findings differ from previous study showing increased IKKβ expression after I/R [50]. Therefore, we detected changes in IKKβ expression in the rat brain at 24, 48, and 72 h after I/R. Results showed that IKKβ expression increased at 24 h and 48 h after I/R, but decreased at 72 h, and was lower than that in the sham group (Fig. 8D, E). Therefore, we speculate that this is a self-protection mechanism of the body.

### MiR-6328 targeted IKKβ expression

**Fig. 9:**
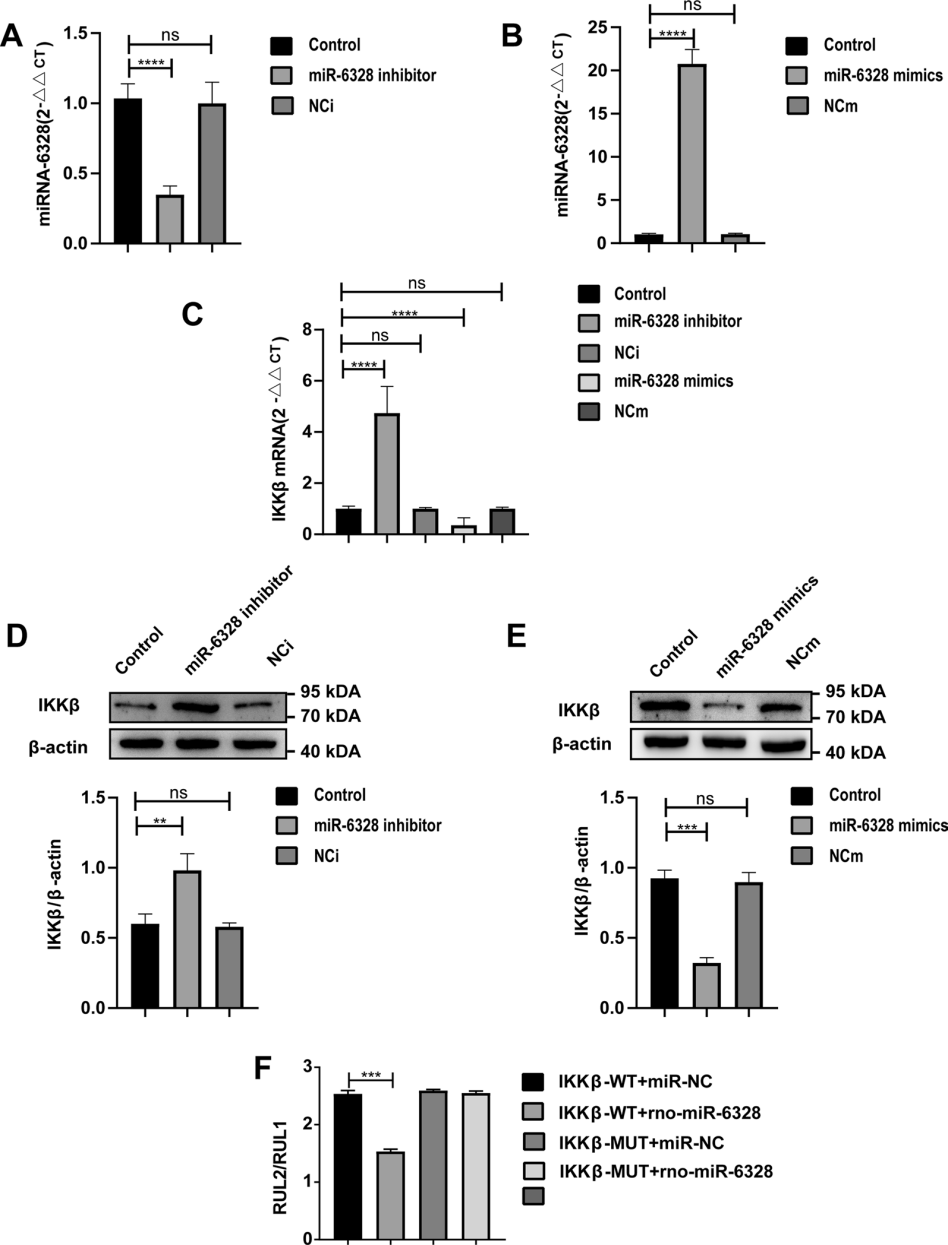
MiR-6328 negatively regulated IKKβ. **A** Expression of miR-6328 in control group and in PC12 cells after transfection of miR-6328 inhibitor and its corresponding negative control NCi. **B** Expression of miR-6328 in control group and in PC12 cells after transfection of miR-6328 mimic and its corresponding negative control NCm. **C** Expression of IKKβ at the mRNA level in control group and expression of IKKβ mRNA in PC12 cells after transfection of miR-6328 inhibitor, NCi, miR-6328 mimic, and NCm. **D** Expression of IKKβ in control group and expression of IKKβ protein in PC12 cells after transfection of miR-6328 inhibitor and its corresponding negative control NCi. **E** Expression of IKKβ in control group and expression of IKKβ protein in PC12 cells after transfection of miR-6328 mimic and its corresponding negative control NCm. **F** Dual-luciferase reporter shows that miR-6328 negatively regulated expression of IKKβ. Data are presented as means ± SEM. ^ns^*P* ≥ 0.05. **P < 0.01, ***P < 0.001, ****P < 0.0001

To verify the targeting relationship between miR-6328 and IKKβ, a miR-6328 inhibitor and miR-6328 mimic were co-cultured with PC12 cells to detect changes in miR-6328 and IKKβ expression. Results showed that miR-6328 expression in the PC12 cells was decreased after miR-6328 inhibitor pretreatment and increased after miR-6328 mimic pretreatment (Fig. 9A, B). When miR-6328 was inhibited, IKKβ expression was significantly increased, whereas when miR-6328 was overexpressed, IKKβ expression was significantly inhibited (Fig. 9C–E). The dual-luciferase reporter results showed that miR-6328 a certain targeting (negative regulation) relationship with IKKβ (*P* < 0.001; Fig. 9F).

### NP1 reduced expression of IKKβ and phosphorylation levels of NF-κB p65 and I-κB in rat brain at 48 h after I/R injury

**Fig. 10:**
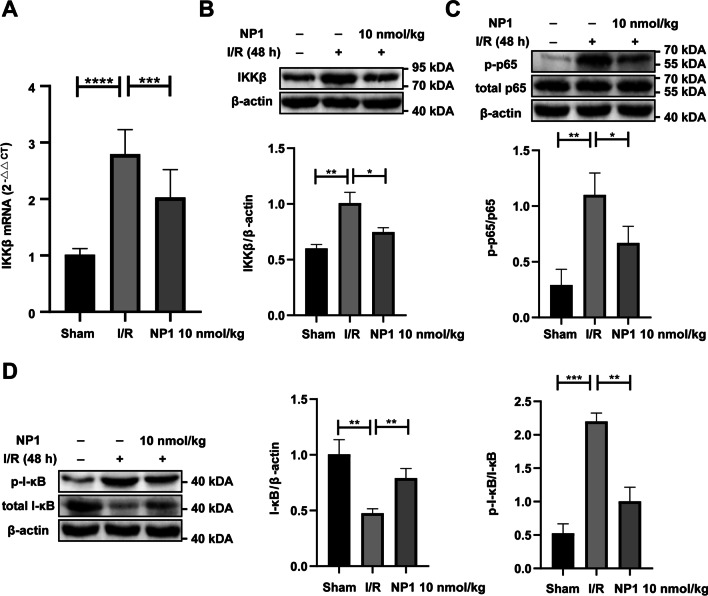
NP1 reduced IKKβ and inhibited NF-κB activation at 48 h after I/R (*n* = 3). **A** NP1 reduced expression of IKKβ at the mRNA level at 48 h after I/R injury in rats. **B** NP1 reduced expression of IKKβ at the protein level at 48 h after I/R injury in rats. **C** NP1 reduced phosphorylation levels of NF-κB p65 at 48 h after I/R injury in rats. **D** NP1 increased expression of total I-κB protein and reduced phosphorylation levels of I-κB at 48 h after I/R injury in rats. Data are presented as means ± SEM. *P < 0.05, **P < 0.01, ***P < 0.001, ****P < 0.0001

The I-κB kinase complex IKK is a core factor regulating the NF-κB signaling pathway and plays a crucial role in the progression of cerebral I/R disease [51, 52]. After I/R injury, I-κB and NF-κB p65 are phosphorylated by IKKβ and total I-κB protein is rapidly degraded, which activates the NF-κB signaling pathway to exacerbate inflammation and I/R-induced damage [53]. Here, NF-κB pathway activation in the brains of rats in each group was verified by detecting the phosphorylation levels of I-κB and NF-κB p65. As shown in Fig. 8D, E, IKKβ expression increased most significantly 48 h after I/R, and the protective effect of NP1 was more evident at this time. Therefore, the impact of NP1 on IKKβ expression and NF-κB pathway activation was detected 48 h after I/R. As shown in Fig. 10A, B, NP1 effectively reduced the increased expression of IKKβ at 48 h after I/R. The phosphorylation levels of NF-κB p65 and I-κB were significantly increased and total protein content of I-κB was significantly decreased at 48 h after I/R, whereas NP1 (10 nmol/kg) treatment effectively reversed this trend (Fig. 10C, D). These results indicated that NF-κB p65 and I-κB phosphorylation levels were significantly decreased after NP1 treatment (10 nmol/kg), which inhibited NF-κB pathway activation and reduced damage caused by I/R.

**Fig. 11:**
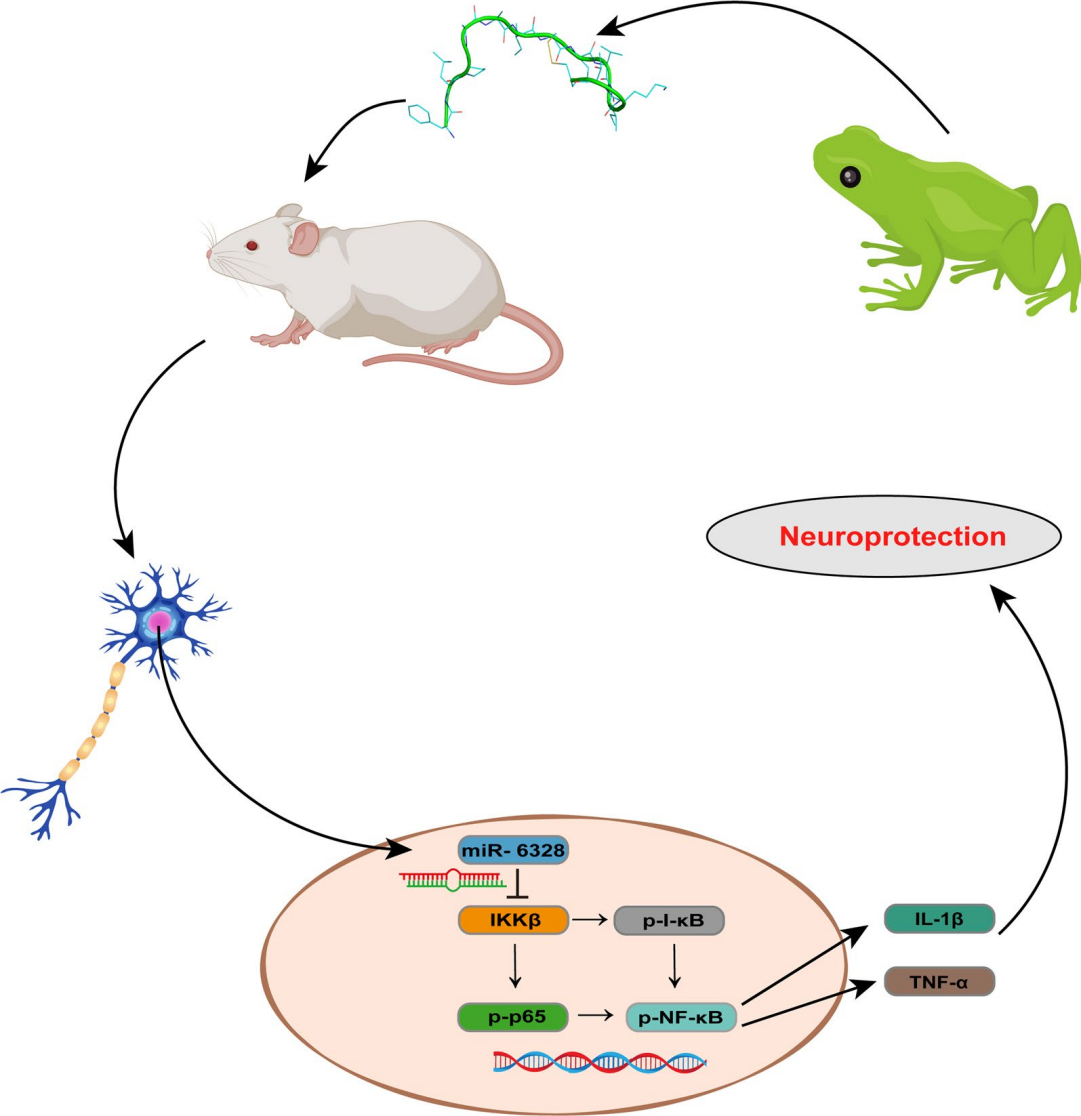
Hypothesis map of neuroprotective effects of NP1

Based on the above research, we speculated that the neuroprotective role of NP1 in cerebral I/R damage is exerted by up-regulating miR-6328 to reduce the expression of IKKβ, thereby inhibiting activation of the NF-κB pathway (Fig. 11).

## Discussion

The newly identified neuroprotective peptide NP1, extracted from the skin of *R. limnocharis*, contained 13 amino acids (Fig. 1A). Compared with previously reported natural peptides with anti-stroke properties, e.g., OM-LV20 [54], Exendin-4 [33], and sylvestin [55], NP1 has a shorter amino acid sequence, lower molecular weight, and a pair of intramolecular disulfide bonds (Fig. 1B). Thus, NP1 is cheaper to synthesize and more easily absorbed by the body, with the disulfide bonds increasing its stability and decreasing its decomposibility [56]. NP1 demonstrated biosafety in vivo and in vitro. Notably, it showed no acute toxicity or hemolytic activity and did not cause damage to the main organs of the rats (Tables 1, 2; Fig. 2), highlighting its superiority. Furthermore, with the above characteristics, NP1 successfully crossed the blood–brain barrier (Fig. 3) and showed superior neuroprotective and anti-stroke activities. Thus, NP1 was selected to study IS induced by I/R injury using the MCAO/R model in rats. NP1 exerted moderate protection against I/R injury in rats at doses of 0.1 and 1 nmol/kg but marked neuroprotective effects at 10 nmol/kg (Fig. 4). Intraperitoneal injection also provides a convenient application approach for treatment. Notably, NP1 reduced cerebral infarct size, relieved neurological dysfunction, reduced brain cell damage caused by I/R, and increased neuronal survival in rats 72 h after I/R (Figs. 4, 5). NP1 also reduced PC12 cell death after OGD/R injury in vitro (Fig. 6E).

The pathophysiological events involved in IS development are complex, and the onset of inflammation is highly damaging [57]. Studies have shown that the expression levels of TNF-α, IL-1β, and other inflammation-related factors peak after 3 days of ischemic encephalopathy [58]. Thus, we sampled rats after 72 h of I/R. Western blotting showed that NP1 reduced the expression levels of TNF-α and IL-1β in vitro and in vivo (Fig. 6A–D, F–I). However, the regulation of TNF-α and IL-1β expression by NP1 was still unclear and was therefore further explored.

MiRNAs, which play critical roles in regulating stroke progression, are potential drug targets for the treatment of IS [59]. In recent years, miRNAs have gained increasing attention as powerful drug targets. However, there are few reports on the role of peptide drugs in ischemic encephalopathy via miRNA modulation. Therefore, miRNA sequencing was used to elucidate the neuroprotective mechanism associated with the anti-stroke properties of NP1 (Fig. 7). Through further analysis of the sequencing results, miR-6328 and IKKβ were selected for follow-up exploration. IKKβ promotes the activation of NF-κB [52], which regulates many physiological and pathological processes, such as inflammation and apoptosis, and plays an important role in IS [51]. In the resting state, IKKβ and NF-κB p65 exist in the cytoplasm in inactive forms. However, when IKKβ is activated by brain injury, it rapidly phosphorylates and degrades I-κB to activate NF-κB p65, resulting in nuclear displacement, NF-κB p65 phosphorylation, and NF-κB pathway activation [52], which aggravate the inflammatory response by increasing the release of IL-1β and TNF-α [60]. Based on verification of the sequencing results, we found that miR-6328 expression increased, while IKKβ expression decreased 72 h after I/R injury, and NP1 further reduced the expression of IKKβ in the cerebral cortex of rats after I/R (Fig. 8A–C). Furthermore, NP1 regulated the expression of IKKβ by targeting miR-6328 (Fig. 9). As the increasing trend of IKKβ was most obvious at 48 h after I/R (Fig. 8D, E), we further analyzed the expression of IKKβ and phosphorylation of NF-κB p65 and I-κB at this time point to clarify the neuroprotective effects of NP1. Results showed that the expression of IKKβ and phosphorylation of NF-κB p65 and I-κB were significantly increased after 48 h of I/R, and the NF-κB pathway was activated at this time point. However, NP1 intervention effectively alleviated this trend (Fig. 10). Thus, we speculated that NP1 inhibited I-κB and NF-κB p65 phosphorylation by mediating IKKβ inhibition by miR-6328 and alleviated inflammation after I/R in rats.

Our results showed that miR-6328 expression increased while IKKβ expression decreased 72 h after I/R injury (Fig. 8A–C, inconsistent with previous research [50]. Several studies have shown that, despite causing injury, inflammation also maintains the balance of physiological responses in the body [61]. In addition, while certain neuroinflammatory pathways are destructive, they can also repair cerebral ischemic damage [62]. After I/R injury, inflammation typically occurs in three phases: i.e., acute, subacute, and chronic phases. Although the expression of inflammatory factors and activation of pathways differ in the three phases, how expression changes is not yet clear [61]. Here, we found that IKKβ expression increased at 24 h and 48 h after I/R injury but decreased to below normal levels after 72 h (Fig. 8D, E). We speculate that this may be a form of self-protection, although the underlying mechanism remains unclear and requires further study. In addition, according to KEGG enrichment analysis (Fig. 7D), the role of NP1 in inhibiting the MAPK pathway and activating the PI3K/Akt pathway will be clarified in future studies to understand the neuroprotective effects and potential mechanisms of NP1.

## Conclusions

The newly discovered neuroprotective peptide NP1 obtained from amphibian skin secretions demonstrated powerful neuroprotective effects by inhibiting the NF-κB pathway via miR-6328 targeting IKKβ. Thus, this study provides a potential new drug and drug target for the treatment of IS.

## Data Availability

All data supporting this study are available from the corresponding author upon reasonable request.

## Abbreviations

Faslg: Fas ligand
Fgfr2: Fibroblast growth factor receptor 2
GO: Gene ontology
I/R: Ischemia–reperfusion
IL-1β: Interleukin-1β
IL-8: Interleukin-8
IL-6: Interleukin-6
IRS1: Recombinant insulin receptor substrate 1
MAP3K5: Apoptosis signal-regulating kinase 1
MAP3K3: Mitogen-activated protein kinase/extracellular signal-regulated kinase kinase kinase 3
MAPK1: Mitogen-activated protein kinase 1
I-κB: Inhibitor of NF-κB
IKKβ: Inhibitor kappa B kinase β
KEGG: Kyoto Encyclopedia of Genes and Genomes
MCAO: Middle cerebral artery occlusion/reperfusion
MAPK: Mitogen-activated protein kinase
NP1: Neuroprotective peptide 1
NF-κB: Nuclear factor-kappa B
OGD/R: Oxygen–glucose deprivation/re-oxygenation
PI3K/AKT: Phosphatidylinositol 3-kinase/protein kinase B
PIK3R2: Phosphoinositide 3-kinase regulatory subunit 2
qRT-PCR: Quantitative real-time polymerase chain reaction
rt-PA: Recombinant tissue plasminogen activator
Rela: Nuclear factor-kappa B p65
SD: Sprague–Dawley
TNF-α: Tumor necrosis factor-α
TTC: 2,3,5-Triphenyltetrazolium chloride
